# Current Perspectives on Non-Metastatic Male Breast Cancer: Genetics, Biology, and Treatment Advances: A Systematic Review

**DOI:** 10.3390/cancers17193270

**Published:** 2025-10-09

**Authors:** Kathleen Melan, Pierre Loap, Youlia Kirova

**Affiliations:** 1 Department of Radiation Oncology, Institut Curie, 75005 Paris, France; 2 Department of Radiation Oncology, University Hospital of Martinique, 97200 Fort-de-France, Martinique, France

**Keywords:** male breast cancer, genetics, tumor biology, treatments, endocrine therapy, survival

## Abstract

**Background/Objectives:** Male breast cancer (MBC) is a rare malignancy representing less than 1% of all breast cancer cases, with rising incidence worldwide. Current treatment strategies largely rely on extrapolation from female breast cancer, despite clear biological and clinical distinctions. This review aims to summarize current knowledge on non-metastatic MBC, with a particular focus on genetic predisposition, tumor biology, and recent therapeutic advances. **Methods:** A systematic literature search was conducted using PubMed and PMC databases to identify clinical trials, observational studies and systematic reviews related to MBC published up to 1st June, 2025. Studies were selected for their relevance to genetic and molecular features, as well as treatment outcomes in non-metastatic disease. **Results:** Fifty-one studies were included in the review. Findings confirm the predominance of hormone receptor–positive tumors in MBC and underscore the central role of ***BRCA2*** mutations. Germline mutations in ***BRCA2*** and ***BRCA1*** were reported in approximately 1 and 2% of male cases, respectively. Additional germline alterations were identified in ***PALB2*, *CHEK2***, and other DNA repair genes. Comparative analyses of surgical approaches showed no significant difference in survival between breast-conserving surgery and mastectomy. Postmastectomy radiotherapy improved overall survival compared to surgery alone. Adjuvant tamoxifen therapy was independently associated with significant survival benefits, although adherence remains a challenge. **Conclusions:** MBC is a biologically distinct and molecularly heterogeneous disease. Breast-conserving surgery appears safe and effective in selected patients. Adjuvant radiotherapy and tamoxifen confer clear survival advantages. The lack of male-specific clinical trials remains a major limitation in optimizing evidence-based care for MBC.

## Simple Summary

Male breast cancer is a rare disease with unique genetic and biological characteristics that differ from female breast cancer. This review summarizes the current understanding of genetic predisposition, molecular features, and clinical behavior in men, highlighting key genes such as *BRCA2*, *CHEK2*, and *PALB2*, and describing differences in hormone receptor expression and tumor biology. We also examine advances in treatment, including breast-conserving surgery, sentinel lymph node biopsy, radiotherapy, and endocrine therapy, emphasizing strategies that provide effective cancer control while minimizing side effects. By integrating genetic, molecular, and clinical data, this work aims to improve awareness among researchers and clinicians, guide personalized treatment, and identify areas for future investigation. The findings may help optimize diagnosis, management, and long-term outcomes for men with breast cancer, ultimately bridging gaps in knowledge that have historically focused on female patients.

## 1. Introduction

Male breast cancer (MBC) remains a rare disease, accounting for less than 1% of all breast cancer diagnoses worldwide [1,2]. However, this rarity conceals significant biological complexity and a sharply increasing global incidence, highlighting an urgent and specific public health challenge. In France, the incidence is estimated at approximately 1 case per 100,000 men, a figure stable over recent years, though the absolute number of new cases rises with demographic aging [3]. Globally, recent data from GLOBOCAN and the 2021 Global Burden of Disease (GBD) Study confirm a significant increase in MBC incidence since 1990 [1,2]. Furthermore, marked ethnic disparities exist, notably in the United States where Black men have up to a 52% higher incidence compared to White men [4].

From a clinical and research perspective, non-metastatic MBC constitutes a distinct and critical entity: it corresponds to the stage where tumors remain localized or regionally confined, and where therapeutic interventions—surgery, radiotherapy, and systemic adjuvant treatments—still aim at long-term remission or cure.

Historically, MBC treatment protocols have largely been adapted from those for female breast cancer (FBC) [5], overshadowing critical sex-related differences in tumor genetics, hormonal context, and clinical behavior. This systematic reliance on extrapolation results in suboptimal management of a disease that is both molecularly distinct and clinically heterogeneous.

The prognosis of MBC often remains poor, driven by frequently late diagnosis [6]. Although overall survival is gradually improving, it remains inferior to that observed in women. One barrier to optimizing treatment is the frequent exclusion of men from breast cancer clinical trials, which limits the availability of male-specific data [7]. Consequently, guidelines from organizations such as the National Comprehensive Cancer Network (NCCN), the European Society for Medical Oncology (ESMO), and the American Society of Clinical Oncology (ASCO) still provide limited male-specific recommendations [5,8,9].

In France, the systematic implementation of genetic testing for all male patients now allows a detailed exploration of the unique genetic characteristics of male breast cancer, offering unprecedented potential for early detection and personalized treatments.

Despite significant progress in the understanding and treatment of female breast cancer, knowledge regarding MBC remains scarce. The rarity of this malignancy has long hindered the establishment of robust cohorts and the conduct of dedicated clinical trials, leading to a fragmented view of its biology and clinical course. Most available evidence derives from small retrospective series or extrapolation from female data, which continues to perpetuate suboptimal management.

This situation reveals a significant gap in the understanding and management of MBC. A dedicated and systematic approach is therefore essential to address these shortcomings. Focusing specifically on non-metastatic MBC is particularly relevant, as this stage represents the setting where multidisciplinary interventions—including surgery, radiotherapy, and systemic therapies—offer the greatest potential for curative outcomes. Investigating its biological mechanisms and treatment strategies therefore has direct translational value and important health economic implications. From a methodological perspective, restricting the scope to non-metastatic disease allows a clearer and more consistent evaluation of prognostic and therapeutic factors, avoiding the added complexity introduced by advanced metastatic disease.

This review synthesizes the latest advances in genetic underpinnings, tumor biology, and evolving therapeutic strategies for non-metastatic male breast cancer, providing a critical update aimed at catalyzing future research and improving patient-centered care in this neglected population.

## 2. Materials and Methods

This systematic review was conducted using a structured methodology in accordance with PRISMA guidelines with the objective of synthesizing available data on genetics, molecular biology, and treatment advances in non-metastatic male breast cancer (MBC). A comprehensive search was performed in PubMed and PubMed Central (PMC), which were selected because they index the majority of peer-reviewed biomedical journals relevant to oncology and breast cancer. The search covered all studies published up to 30 June 2025. To maximize sensitivity, both Medical Subject Headings (MeSH) and free-text terms were used in combination, including “male breast cancer,” “male breast neoplasms,” “non-metastatic,” “early-stage,” “localized,” “genetics,” “biology,” “germline variants,” “treatment,” “management,” “endocrine therapy,” “chemotherapy,” “radiotherapy,” “surgery,” and “survival.” Boolean operators (AND, OR) were applied to combine these terms, and the reference lists of relevant reviews and original studies were manually screened to identify additional publications.

Studies were eligible if they reported original data on male breast cancer, specifically focusing on non-metastatic disease or providing extractable information on this subgroup, and if they included findings related to genetics, molecular biology, or treatment strategies such as surgery, radiotherapy, endocrine therapy, or systemic therapy. We excluded single case reports with fewer than five patients, studies not reporting sex-specific outcomes, and articles not available in English or French. Editorials, commentaries, letters, and studies focusing exclusively on metastatic disease were also excluded.

The selection process was conducted in two stages: titles and abstracts were first screened to assess relevance, followed by full-text review of potentially eligible articles. Two reviewers independently assessed study eligibility, and discrepancies were resolved by consensus. Data extracted from the final set of studies included study design, patient population, sample size, genetic and biological findings, treatment modalities, and clinical outcomes where available.

**Figure 1:**
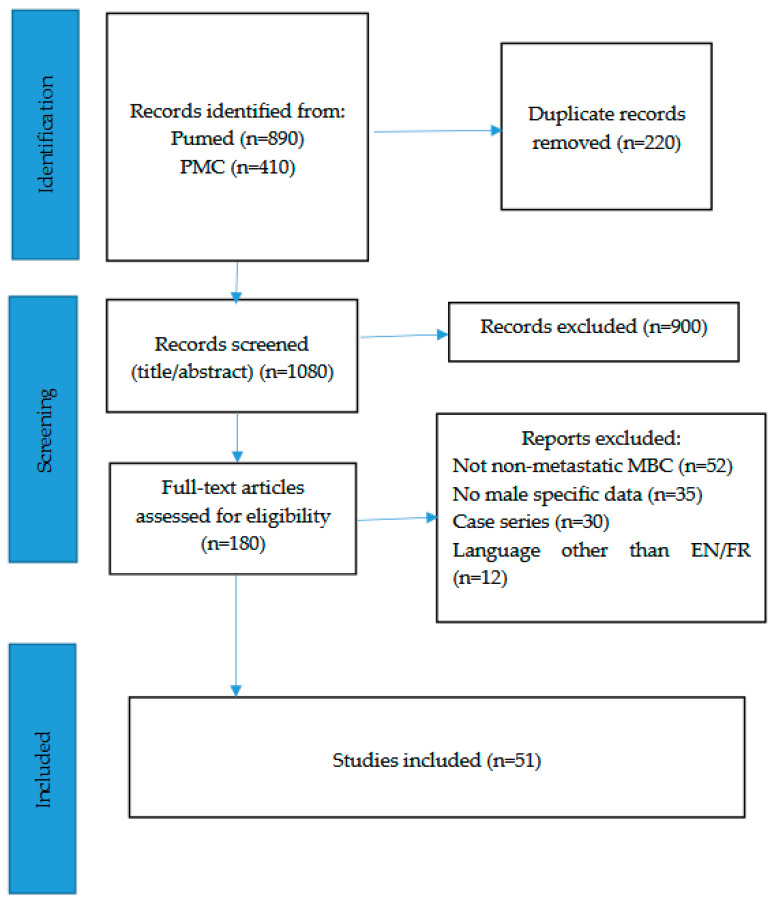
PRISMA flow diagram.

The initial search identified a total of 1300 records (PubMed: 890; PMC: 410). After removal of 220 duplicates, 1080 records were screened by title and abstract, of which 900 were excluded as irrelevant. The full texts of 180 articles were then assessed for eligibility, resulting in 51 studies being included in this review. The overall selection process is summarized in a flow diagram (Figure 1), which illustrates the number of records identified, screened, assessed, and included.

## 3. Results

### 3.1. Genetic Alterations Shaping Male Breast Cancer Risk

**Figure 2:**
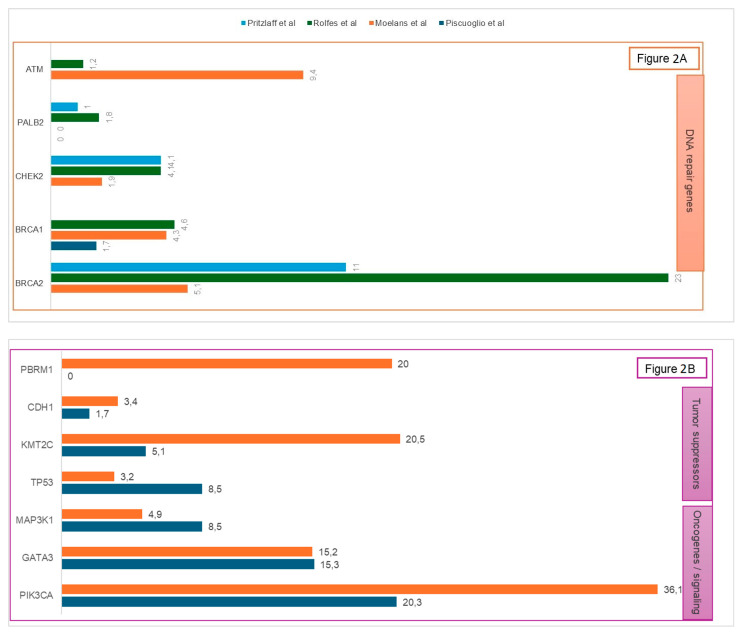
Frequency of germline (**A**) and somatic (**B**) genetic alterations in male breast cancer across major published series (%).

Hereditary germline variants were identified in 10–20% of MBC, with up to one-third of cases occurring in families with a history of breast and/or ovarian cancer [10,11]. Figure 2 highlights the genetic heterogeneity in male breast cancer. Susceptibility genes can be stratified according to penetrance, ranging from rare, high-risk variants to common, low-effect alleles (Table 1).

#### 3.1.1. High-Penetrance Genes

Hereditary germline variants of *BRCA1* and *BRCA2* account for approximately 2% and 10% of male breast cancer (MBC) cases, respectively, and the lifetime risk of developing breast cancer for men carrying *BRCA1* and *BRCA2* mutations is estimated at 1.2% and 6.8% [14,15]. In analyses of familial cohorts, *BRCA2* predominates, representing 60–76% of high-risk familial cases, compared with 10–16% for *BRCA1* [13,16,17,18]. Founder populations, such as Ashkenazi Jews, show a prevalence of 13% for *BRCA2 6174delT mutations* [18].

Unlike female breast cancers (FBC), where certain *BRCA2* regions, such as the Ovarian Cancer Cluster Region (OCCR), are associated with site-specific cancer risks, no region-specific correlation has been observed in men [14]. However, some truncating and founder mutations, such as *BRCA2 9346(-2) A>G*, are more frequent in men, suggesting a sex-related genotypic bias [15,19]. Large genomic rearrangements (>500 kb) are also more frequent in *BRCA2* than in *BRCA1* [20]. Frequent somatic alterations, particularly loss of heterozygosity and somatic *BRCA2* mutations, further support the key role of this gene in male tumorigenesis.

In contrast, *BRCA1* mutations confer a moderate risk (1–2%) in men, with no clear correlation with methylation, loss of heterozygosity, or specific tumor phenotype [21]. These data suggest that while *BRCA1* may contribute to male predisposition in certain subgroups, sex-specific risk-modifying factors remain unknown. The Ashkenazi founder mutations *BRCA1 185delAG* and 5382insC remain notable [18,21].

Beyond *BRCA1* and *BRCA2*, Wen et al. [22] identified pathogenic or likely pathogenic germline variants in *BARD1, ATR, BRIP1*, and *CHEK2* in *BRCA1/2*-negative men, increasing the overall diagnostic yield of multigene panels from 12.5% to 33% in MBC. Integration of these variants with a polygenic risk score showed a significant correlation with early-onset MBC (*p* = 1.10 × 10^−4^), suggesting an interplay between monogenic and polygenic predisposition.

#### 3.1.2. Moderate-Penetrance Genes

Variants in *CHEK2*, *PALB2*, and *ATM* have been consistently associated with increased MBC risk. The recurrent *CHEK2 c.1100delC* variant confers a four- to tenfold increased risk and accounts for up to 9% of high-risk families [12,23]. However, prevalence varies: some *BRCAX* families show frequencies up to 13.5%, while unselected cohorts show no enrichment, indicating the influence of modifying factors [12].

*PALB2* mutations increase risk approximately fourfold, with consistent enrichment in families with both male and female breast cancer [13,24].

Other Fanconi pathway genes, such as *BRIP1*, *RAD51C*, and *XRCC2*, remain rare or of uncertain contribution; *XRCC2* has been identified in a single early-onset familial MBC case [16]), while FANCM (p.Arg1931*) has been implicated in some Italian cohorts, suggesting a male-specific risk contribution [25]. The role of these genes appears less consistent than that of *CHEK2* or *PALB2* and requires validation in larger studies.

Rare syndromic predispositions, such as those associated with *TP53* (Li-Fraumeni), *PTEN* (Cowden), *STK11* (Peutz-Jeghers), and *MMR genes* (Lynch), have also been reported, but their contribution remains marginal outside the syndromic context [16,25].

The androgen receptor (AR) gene has also been explored. Polymorphic variation in the exon 1 CAG repeat is associated with increased risk and poorer prognosis, although findings are not yet conclusive [26].

#### 3.1.3. Low-Penetrance Alleles and Susceptibility Loci

GWAS have identified several common susceptibility loci associated with increased MBC risk, often overlapping with loci for ER-positive FBC. These include variants at 2q35, 6q25.1 (ESR1), 10q21.2, 11q13.3, 12p11.22, 16q12.1 (TOX3), and 14q24.1 (RAD51B) [27]. Men in the top quintile of polygenic risk had a fourfold higher risk than those in the lowest quintiles (OR = 3.86, 95% CI = 3.07–4.87; *p* = 2.08 × 10^−30^). Certain SNPs, such as rs3803662 (TOX3) and rs1314913 (RAD51B), appear to exert stronger effects in men, highlighting the sex-specific genetic architecture [27].

#### 3.1.4. Somatic Alterations

The most frequent somatic mutations involve *PIK3CA*, with hotspots distinct from FBC. Their frequency is higher in *BRCAX* MBC than in *BRCA2*-mutated MBC (11,18,20). Alterations in *GATA3, EMSY, AR, and CYP17* have also been reported. Unlike FBC, *TP53* mutations are rare in MBC. Data on KRAS are conflicting: historical series reported mutations in up to 25% of cases, whereas recent sequencing studies found negligible frequencies [20,28].

#### 3.1.5. Multigene Panels and Extended Spectrum

The advent of multigene panel testing has expanded the identification of candidate genes. Pathogenic variants in *FANCM, NBN, RECQL*, and other genes have been described among more than 50 genes, although most remain rare [23,25].

#### 3.1.6. Epigenetic Alterations

Comparative genomic hybridization studies in MBC revealed gains at 7q36.1 and 11q13.2 (including *AVEN, PALB2, RSF1, GAB2*), as well as frequent losses of *EGFR, CCND1, BRCA2, PALB2, CHEK1/2, EMSY, CDH1*, and *TP53*. Two distinct subgroups have been observed: a small group with few alterations (“simple MBC”) and a broader group (“complex MBC”) with whole-arm gains and frequent *RB1* losses; gains of *STK11*, *SMARCB*1, and *HRAS* were noted in *BRCA2* mutation carriers [18,20,29].

Methylation profiling of 108 MBCs revealed hypermethylation of at least one tumor suppressor gene in every case (an average of six genes per tumor), including *MSH6, WT1, PAX5, PAX6*, and *CDH13* [30]. *ESR1* and *GSTP1* methylation correlated with a high mitotic index. Compared with FBC, methylation of *ESR1*, *BRCA1*, and *BRCA2* is less frequent in men, whereas *RASSF1A* and *RARβ* show sex-specific differences in methylation and expression [30].

MicroRNA profiling identified 33 upregulated miRNAs (miR-21, miR-519d, miR-183, miR-197, miR-493-5p) and 21 downregulated miRNAs (miR-145, miR-497) in MBC compared with gynecomastia [21].

### 3.2. Molecular Pathways and Clinicopathological Correlates in Male Breast Cancer

#### 3.2.1. Histology and Hormone Receptor Status

Invasive ductal carcinoma is consistently reported as the predominant histological subtype in MBC, accounting for over 85% of cases across all series [29,30,31,32]. Hormone receptor expression is notably high: estrogen receptor (ER) positivity ranges from 74% to 93%, while progesterone receptor (PR) positivity is reported to be between 69% and 84% [29,33]. In the international multicenter patient cohort study by Humphries et al. [31], more than 80% of tumors were ER-positive and over 70% PR-positive. A large French cohort reported ER positivity in 92% of cases [32]. However, a California-based study observed variability by age and ethnicity, with lower ER expression in non-Hispanic Black patients associated with a poorer prognosis [34].

#### 3.2.2. Androgen Receptor (AR) Expression

AR expression is reported in over 70% of male breast cancer cases [18,30,32]. AR expression is frequently co-expressed with ER, although its prognostic value remains debated. Co-expression of ER and AR has been associated with improved prognosis in several studies, although large-scale cohort data remain limited [35,36].

#### 3.2.3. HER2 Overexpression

In contrast to female breast cancer (FBC), the frequency of *HER2* overexpression and gene amplification is substantially lower in MBC. Reported rates generally range between 2% and 5%, which, although much rarer than in FBC, nonetheless define a clinically significant subset of patients with important therapeutic implications. This observation is consistent across several series [31,37,38,39]. Furthermore, the recently recognized category of “*HER2*-low” tumors highlights additional potential for novel targeted therapies. *HER2*-low expression is relatively common in MBC, with reported rates ranging from 27% to nearly 68% depending on cohort and definition, similar to or slightly lower than in female breast cancer [37,38]. *HER2*-low tumors are predominantly hormone receptor–positive and more often diagnosed in older men, with a higher likelihood of lymph node involvement compared to *HER2*-0, but without significant differences in histology, stage, or grade. Approximately 13% of *HER2*-low MBCs harbor germline BRCA1/2 mutations, mainly *BRCA2,* which may have implications for future targeted therapeutic strategies.

#### 3.2.4. Molecular Subtypes

Immunohistochemical classification reveals a predominance of luminal A tumors (65–89%), followed by luminal B (11–29%). *HER2*-enriched and basal-like phenotypes are rare (<10%) [11,18,32,35,40]. Johansson et al. [29] proposed a MBC-specific transcriptomic classification identifying two distinct subgroups: luminal M1, characterized by enrichment in hormone signaling and immune suppression genes, associated with more aggressive tumors and poorer prognosis; and luminal M2, defined by proliferation and metabolic gene signatures, linked to less aggressive disease and better outcomes. These subgroups diverge from the conventional luminal A/B classification used in female breast cancer, highlighting unique molecular features in MBC that may require tailored therapeutic strategies

#### 3.2.5. Tumor Microenvironment and Lipid Metabolism

Single-cell transcriptomic analyses have highlighted a marked activation of fatty acid metabolism (via FASN), reduced T-cell infiltration, and upregulation of p38 MAPK signaling in MBC tumors, indicating active immune evasion [41]. These findings suggest a higher metastatic potential compared to FBC.

#### 3.2.6. Emerging Biomarkers

Several emerging prognostic biomarkers have been identified in MBC. Elevated cytoplasmic expression of DDX3 is significantly associated with improved 10-year overall survival, independently of other clinical factors [29]. ATF3 and FASN are associated with the luminal B phenotype, *BRCA2* mutations, positive nodal status, and a more aggressive tumor biology. In addition, collagen IV has been identified as an independent predictor of recurrence-free survival, underscoring its potential as a prognostic marker [42].

#### 3.2.7. Clinical Presentation and Prognostic Factors

The distinct molecular and genetic features outlined above provide an important framework for re-evaluating clinical management strategies in male breast cancer. Insights such as androgen receptor (AR) overexpression, recurrent *PIK3CA* mutations, and specific characteristics of the tumor microenvironment highlight potential avenues for tailoring therapeutic approaches. Bridging these biological findings with clinical practice is essential to optimize diagnostic pathways, refine prognostic assessment, and guide the development of targeted treatment strategies.

Male patients are generally diagnosed at an older age and at a more advanced stage, with a higher frequency of lymph node involvement compared to female patients [43,44,45]. Poor prognostic factors include nodal involvement, tumor size > 2 cm, and ER-negative status [11,46].

The EORTC 10085/BIG/NABCG international study confirmed that positivity for ER, PR, or AR is significantly associated with better overall and disease-free survival [44]. In the international EORTC 10085/TBCRC/BIG/NABCG study including 1483 male breast cancers, a high mitotic activity index, the presence of a fibrotic focus, and low tumor-infiltrating lymphocyte density were significantly associated with poorer prognosis, whereas overall histological grade was not correlated with survival, in contrast to what is commonly observed in female breast cancer [33]. Hierarchical clustering of 111 male breast cancers identified two distinct IHC-defined subgroups with differing survival, with tumor size, ER status, and metastases as key prognostic factors [40].

In the studies by Kornegoor et al. [30] and Sun et al. [41], *BRCA*-mutated patients, particularly those with *BRCA2,* exhibited a distinct clinical profile—earlier age at diagnosis, larger tumor size, more advanced stage, and poorer overall survival. In Fentiman’s cohort, this subgroup also showed strong associations with more aggressive molecular profiles.

### 3.3. Treatment Advances in Non-Metastatic Male Breast Cancer

#### 3.3.1. Is Breast-Conserving Surgery a Safe and Feasible Alternative to Mastectomy?

Across these datasets, mastectomy remains the predominant surgical approach, reflecting entrenched practice patterns and the limited volume of male breast tissue. Leone et al. [47] analyzed 6919 male breast cancer patients from the SEER database from 1988 to 2017, revealing an overall mastectomy rate of 81,6%. Breast-conserving surgery (BCS) rates ranged from 17% to over 40% in selected institutional series [48,49,50,51,52]. Radiotherapy utilization following BCS varied substantially, from under 50% to nearly systematic use in prospective institutional data [52,53,54].

Nevertheless, both pooled and registry-level analyses consistently demonstrate that breast-conserving surgery (BCS), when combined with adjuvant radiotherapy (BCT), achieves local control and overall survival (OS) outcomes equivalent to mastectomy in appropriately selected patients with early-stage disease. Table 2 presents comparative surgical outcomes and survival endpoints between breast-conserving surgery (BCS) and mastectomy across studies. In pooled analyses from Lin et al. [48], no statistically significant difference in overall survival (OS) was observed between BCS and mastectomy with similar 5-year and 10-year OS rates. (*p* > 0.05). Zaenger et al. [50] reported a significantly higher 5-year cancer-specific survival (CSS) for BCS plus radiotherapy in stage II disease (100% vs. 91.2%, *p* < 0.05). Sauder [52] found no significant differences in disease-free survival (DFS), cancer-specific survival, or OS between the two surgical options reaffirming the oncologic equivalence of both approaches.

De la Cruz et al. [55] similarly reported comparable survival in selected cases, emphasizing the importance of achieving negative margins and delivering radiotherapy. Leone et al. [47] noted that breast cancer-specific survival (BCSS) has remained stable (~84%), while OS improved from 64.6% to 69.1% over the past decades, reflecting advances in multimodal therapy.

Fogh et al. [56] observed that, one year after surgery, tissue fibrosis, arm edema, and reduced range of motion occurred in 13%, 23%, and 27% of patients following modified radical mastectomy; 25%, 0%, and 50% after total skin-sparing mastectomy; and 13%, 0%, and 0% in those undergoing breast-conserving surgery, respectively.

The feasibility and oncologic safety of BCS in men have been further explored by several focused studies. Den et al. [49] reported that BCS can achieve oncologic outcomes equivalent to mastectomy when followed by appropriate radiotherapy, with similar local recurrence rates and DFS between surgical modalities. Giordano [57] noted that while BCS utilization remains low in male breast cancer, its safety profile is comparable to that in females.

Importantly, systematic reviews and institutional studies highlight additional benefits of BCS. De la Cruz et al. [55] found that men undergoing BCS reported high satisfaction with body image and quality of life, a consideration often overlooked in clinical decision-making. Survival outcomes comparing breast-conserving surgery (BCS) and mastectomy at 5 and 10 years are summarized in Table 3.

#### 3.3.2. Axillary Staging: Sentinel Lymph Node Biopsy (SLNB) Performance for Male Breast Cancer

The most comprehensive synthesis to date was provided by Parpex et al. [60], a meta-analysis pooling data from 12 retrospective series. This analysis included 164 patients for sentinel node identification rate (IR) and 50 patients for false-negative rate (FNR) assessment. The pooled IR was 99.0%, and the pooled FNR was 0%, with no heterogeneity reported. These results confirm the high diagnostic reliability of SLNB in MBC, comparable to benchmarks observed in female cohorts.

Several individual institutional series reinforce these findings. Additionally, an umbrella review [61], reported a pooled identification rate and a false-negative rate closely aligned with benchmark values in female breast cancer. These data support SLNB as the preferred axillary staging technique, reserving axillary lymph node dissection for cases with positive SLNB. Table 4 details sentinel lymph node biopsy (performance).

Regarding detection techniques, Şimşek et al. [65] showed that combining blue dye with indocyanine green significantly improved IR compared with blue dye alone (94.9% vs. 83.9%), indicating superior sentinel node identification with the combined approach in male breast cancer.

Recent evidence supports de-escalation of both breast and axillary surgery in selected male patients. Two recent studies further evaluated the omission of complete axillary lymph node dissection (ALND) [66,67]. Yang et al. [66], using a SEER-based cohort, demonstrated that in patients with one to two positive sentinel nodes, omitting ALND was not associated with reduced overall or breast-cancer-specific survival, suggesting that SLNB alone may be sufficient in selected early-stage cases. Indeed, in this cohort of 299 men with T1–T2 tumors and one to two positive nodes treated with adjuvant radiotherapy; they observed a substantial increase in SLNB utilization over time (from 18.8% in 2010 to 61.0% in 2020). Five-year overall survival (OS) and breast cancer-specific survival (BCSS) did not differ significantly between SLNB and ALND, both in the unmatched cohort (OS 78.0% vs. 85.9%; BCSS 91.5% vs. 95.0%) and after propensity score matching (OS 83.9% vs. 82.0%; BCSS 90.1% vs. 96.9%). Multivariable models did not identify axillary surgery type as an independent predictor of survival after adjustment for case-mix. These findings are consistent with the retrospective analysis by Shang et al. [67], which reported reduced postoperative morbidity and lower complication rates with SLNB compared to ALND.

#### 3.3.3. Adjuvant Radiotherapy in Male Breast Cancer

The role of adjuvant radiotherapy (RT) in MBC has been evaluated in multiple retrospective studies and meta-analyses, with variable practice patterns and heterogeneous reporting of target volumes. Jardel et al. [68], in a systematic review of 14 retrospective studies, reported that the proportion of men receiving adjuvant RT ranged widely from 3% to 100%, often with limited specification of irradiated volumes. In stage III patients, post-mastectomy RT was associated with a significant overall survival (OS) benefit, with 10-year OS of 26.4% in irradiated patients versus 11.9% in non-irradiated patients. In a cohort of 1933 men, no significant difference in 5-year OS was observed between irradiated and non-irradiated groups (78% vs. 77%, *p* = 0.371); however, in a matched subgroup of 315 patients, RT significantly improved 5-year OS (83% vs. 54%, *p* < 0.001). Node-positive patients derived the greatest benefit, with 5-year OS of 79% versus 72% (*p* = 0.05) for those with 1–3 positive nodes, and 73% versus 53% (*p* < 0.001) for patients with ≥4 positive nodes.

Lin et al. [48], in a meta-analysis of 15 studies including 11,392 patients (3648 receiving chest wall and/or nodal RT and 7744 undergoing surgery alone), demonstrated significantly improved OS in the RT group, despite a higher proportion of advanced-stage disease. Non-significant trends toward improved 5-year disease-free survival (DFS) and locoregional recurrence-free survival (LRFS) were also noted.

Colciago et al. [69], in a pooled analysis of 80,219 patients, reported a significant reduction in mortality with the addition of RT, with a pooled adjusted hazard ratio (aHR) for OS of 0.73 (95% CI 0.66–0.81).

Despite these benefits, compliance with adjuvant RT remains suboptimal. Sauder et al. [52] reported that only 27–46% of patients undergoing breast-conserving surgery received adjuvant RT, and post-mastectomy RT was delivered in 8–61% of eligible cases. Forster et al. [70] observed that RT was primarily administered after breast-conserving surgery or in node-positive cases, resulting in excellent locoregional control, although dose prescriptions and target volumes varied substantially. Cutuli et al. [71] confirmed significant improvements in locoregional control among node-positive patients, particularly when axillary fields were added to chest wall irradiation.

Long-term retrospective studies further support the survival benefit of adjuvant RT. Eggemann et al. [72], in a 20-year cohort, demonstrated improved OS with post-mastectomy RT, especially in patients with ≥4 positive lymph nodes. Similarly, Asgharian et al. [73], in a 32-year retrospective analysis from Northern Iran, reported 5-year OS rates of 81% in irradiated patients versus 68% in surgery-only patients, accompanied by significantly reduced locoregional recurrence and acceptable toxicity profiles.

#### 3.3.4. Adjuvant Endocrine Treatment: Survival Impact and Compliance Challenges in Male Breast Cancer

A total of 15 studies evaluating adjuvant endocrine therapy (ET) in MBC were included, encompassing both single-institution cohorts and large registry-based analyses. Over the past decades, the use of adjuvant chemotherapy and ET has increased substantially in men. Jardel et al. [68] reported that, in a cohort of 10,965 patients, the proportion of men receiving chemotherapy ranged from 3% to 85% (mean 26%), while ET administration varied from 7% to 92% (mean 45%). In a subset of 489 MBC patients treated between 1990 and 2005, 72% received ET—primarily tamoxifen (85%) and aromatase inhibitors (12%)—with uptake rising from 57% in 1988–1995 to 82% in 1996–2005 (*p* < 0.0001). Adjuvant chemotherapy (mostly anthracycline-based) was delivered to 34% of patients, increasing from 25% to 37% over the same period (*p* = 0.029).

Rutherford et al. [74], analyzing 39,529 men with predominantly ER-positive disease (84%), reported heterogeneous ET administration, with a mean treatment rate of 58%. Lin et al. [48] pooled data from ten cohort studies including 11,229 patients and confirmed that most men had hormone receptor-positive tumors, while few were *HER2*-positive. In this meta-analysis, tamoxifen therapy conferred a substantial benefit, improving both disease-free survival (HR 0.44, 95% CI 0.28–0.69) and overall survival (HR 0.62, 95% CI 0.41–0.95) compared with patients who did not receive adjuvant tamoxifen. Five-year OS was increased (OR 1.76, 95% CI 1.60–1.94) as well as 10-year OS (OR 1.87, 95% CI 0.98–3.54). Similarly, 5-year and 10-year DFS were improved (OR 2.72, 95% CI 1.57–4.70; OR 3.34, 95% CI 1.95–5.71, respectively). Comparative analyses showed superior 5-year OS for tamoxifen versus aromatase inhibitor alone (OR 2.35, 95% CI 1.17–4.74), while the combination of aromatase inhibitor with gonadotropin-releasing hormone (GnRH) analogues in metastatic MBC was favored over aromatase inhibitor monotherapy (OS OR 2.40, 95% CI 0.83–6.97).

Recent studies further highlighted both efficacy and challenges in adherence. Yadav et al. [75] reported that in a large cohort of 257 men receiving adjuvant ET, tamoxifen use was associated with a significant improvement in overall survival (HR 0.48, 95% CI 0.30–0.77) and disease-free survival (HR 0.52, 95% CI 0.33–0.82). Their analysis also highlighted that adherence remains a major challenge, with 25% of patients discontinuing therapy within 2 years due to adverse events, including hot flashes, decreased libido, and thromboembolic complications. Venigalla et al. [76] and Popa-Nimigean et al. [77] confirmed that poor compliance was consistently associated with higher recurrence rates and inferior long-term survival, with 20–30% of men discontinuing therapy within two years. These data collectively underscore that adjuvant ET, particularly tamoxifen, confers a substantial survival advantage in MBC, yet its real-world effectiveness is limited by adherence and tolerability issues.

## 4. Discussion

MBC is increasingly recognized as a biologically distinct entity from female breast cancer, exhibiting both overlapping and divergent molecular characteristics. Our results confirm the central role of germline *BRCA2* mutations, representing the most significant high-penetrance gene [78,79]. In contrast, *BRCA1* appears to play a limited role, with a distinctive profile lacking loss of heterozygosity or promoter methylation, suggesting sex-specific predisposition mechanisms. These observations highlight the importance of considering sex as a modulator of mutation penetrance and phenotype, with implications for genetic counseling and familial screening.

Beyond high-penetrance genes, moderate-penetrance variants, such as *CHEK2* and *PALB2,* confer substantial risk, particularly in high-risk families. The identification of additional variants in Fanconi pathway genes (*BRIP1, RAD51C, XRCC2, FANCM*) and in rare syndromes suggests a complex genetic architecture, combining monogenic and polygenic contributions. Integrating polygenic risk scores into MBC analyses could improve risk stratification, particularly for *BRCA1/2*-negative patients, and guide surveillance and prophylactic strategies [80]. The detection of other mutations highlights the need for broader genomic approaches, such as panel or whole-exome sequencing, to better characterize genetic profiles and tailor treatment strategies accordingly. Extended multigene panels are now capable of identifying an increasing proportion of pathogenic variants, with implications for familial screening, prevention, individual risk assessment, and therapeutic decisions, including PARP inhibitor prescription for *BRCA2* carriers. However, uncertainty regarding the variable penetrance of moderately associated genes and the impact of rare variants still limits the precision of clinical recommendations.

Somatic analyses indicate frequent alterations in PIK3CA and GATA3, while TP53 mutations remain rare, contrasting sharply with FBC profiles. This distinction may reflect MBC-specific oncogenic pathways, with potential implications for targeted therapies. Differences in mutational hotspots and the higher frequency of somatic alterations in *BRCA*X MBC compared to *BRCA2* carriers suggest germline–somatic interactions warranting further investigation.

Epigenetic mechanisms also appear to play a critical role in MBC. Reduced methylation of genes such as ESR1 and *BRCA1/2,* along with a distinct miRNA profile, indicate MBC-specific signatures that may influence both tumorigenesis and prognosis. Classification into “simple” and “complex” MBC subgroups reflects notable tumor heterogeneity, which could inform future therapeutic selection.

In France, current recommendations advise systematic genetic counseling for all men diagnosed with breast cancer before age 71, and after 71 in the presence of a suggestive family history [3]. From a translational perspective, these findings support the use of multigene panels and integrated strategies combining genetic, transcriptomic, and epigenetic data for risk stratification and therapeutic planning. They also highlight the importance of investigating interactions between genetic factors and the tumor microenvironment to better understand biological and clinical differences between MBC and FBC.

Management of male carriers of pathogenic germline variants remains poorly defined. In the presymptomatic setting, available evidence—mainly from studies by Campos, Pensabene, and Rolfes included in this review—suggests that men carrying breast cancer–associated variants (notably BRCA1, BRCA2, PALB2, CHEK2, ATM) should be offered genetic counseling, annual clinical breast examination starting at age 35, and annual mammography of the contralateral breast after treatment of a primary tumor or in the presence of a high-risk mutation. Routine MRI screening is not recommended, whereas surveillance should also include assessment of the risk of other associated malignancies (e.g., prostate and pancreatic cancer), depending on the implicated gene. After a diagnosis of male breast cancer, follow-up strategies remain even less standardized, with no consensus on the optimal frequency or modality. While some experts advocate clinical monitoring and selective imaging guided by symptoms or risk profile, prospective data are virtually absent. This ongoing controversy highlights the unmet need for dedicated guidelines and prospective studies to better inform surveillance in this rare population.

A subject of great interest to the clinical community is the management of transgender individuals with germline variants or a history of gender-affirming interventions. In transgender females, long-term estrogen exposure appears to increase breast cancer risk compared to cisgender men, though it remains lower than in cisgender women, with most tumors being luminal and hormone receptor-positive. In transgender males, the risk is reduced after chest-contouring surgery but not eliminated, as residual tissue may persist, and the impact of testosterone therapy remains uncertain. Screening must be tailored: clinical vigilance after mastectomy and adherence to female protocols in those without surgery. Treatment follows standard protocols, but continuation of gender-affirming hormones should be discussed in a multidisciplinary setting. Prospective data and dedicated guidelines are urgently needed to optimize care in this population.

While these specific scenarios illustrate the complexity of managing subgroups with unique risk profiles, the broader challenge remains to integrate emerging biological insights into the routine clinical management of non-metastatic male breast cancer. The distinct molecular features described above provide a new perspective for re-evaluating clinical management strategies in MBC. High rates of hormone receptor and AR expression, the presence of actionable alterations such as *BRCA2* and *PIK3CA*, and distinct tumor–microenvironment interactions support a more biology-driven approach to treatment selection. These insights directly inform the discussion of surgical, radiotherapeutic, and systemic management that follows.

Genetic testing recommendations for male breast cancer are now largely harmonized across international guidelines. Both NCCN and ASCO endorse universal germline testing for all affected men, irrespective of age, family history, or histology, with emphasis on *BRCA1/2, PALB2, CHEK2*, and other susceptibility genes. ERN GENTURIS similarly supports broad access to multigene panel testing and highlights the importance of genetic counseling for patients and relatives. This consensus reflects the relatively high prevalence of germline mutations in MBC—estimated at 15–20%, predominantly BRCA2—and the limitations of restrictive criteria that risk under-diagnosis. Collectively, systematic genetic testing has emerged as a cornerstone of precision management and familial risk prevention in male breast cancer.

Epidemiological risk for male breast cancer is shaped by age, family history, and germline predisposition, particularly *BRCA2*, with smaller contributions from *BRCA1* and rarer genes such as *PALB2, CHEK2, ATM, TP53*, and *PTEN*. Additional risk factors include Klinefelter syndrome, conditions linked to increased lifetime estrogen exposure, prior chest irradiation, and exogenous hormones or antiandrogens. Commonly used risk calculators, including Gail and Tyrer-Cuzick/IBIS, were developed for women and are not validated in men, while CanRisk (BOADICEA) allows male family history entry but remains poorly tested in male populations. Recently, male-specific nomograms incorporating clinical variables such as age, receptor status, and tumor stage have shown promising predictive performance [81,82]. Pending the development of robust, validated male-specific tools, risk assessment should rely on genetic counseling and testing in relevant cases, supported by individualized surveillance strategies.

Histopathological analyses complement this genetic landscape. Invasive ductal carcinoma remains predominant, with high expression of ER and PR, consistent with luminal A/B subtypes [44]. Frequent AR co-expression suggests integrated hormonal regulation, supporting exploration of combined endocrine and AR-targeted therapeutic strategies [73,74]. Transcriptomic and immunohistochemical studies reveal a male-specific tumor microenvironment characterized by activated lipid metabolism, reduced T-cell infiltration, and inhibitory tumor–T-cell interactions. Emerging biomarkers such as FASN, ATF3, and DDX3 show prognostic potential, although clinical validation is still required A. These recent data emphasize the importance of considering male-specific biological and genetic features, suggesting that tailored therapeutic strategies could improve outcomes and quality of life.

Clinically, mastectomy remains the primary local treatment, often justified by the small male breast volume and the frequent retroareolar location of tumors [77,83]. However, recent multicenter cohorts and meta-analyses confirm the oncologic safety of breast-conserving surgery (BCT) with adjuvant radiotherapy in selected early-stage patients [61]. Although adoption is limited by small breast volume, aesthetic benefits and body image satisfaction provide additional rationale for its use in appropriate cases. Sentinel lymph node biopsy (SLNB) demonstrates high identification rates (>97%) with negligible false negatives, allowing complete axillary dissection to be reserved for node-positive cases [60,84].

Adjuvant radiotherapy improves locoregional control and survival, particularly in node-positive patients. Multicenter series and meta-analyses report significant improvements in overall and recurrence-free survival with acceptable toxicity. Nevertheless, real-world use of radiotherapy remains heterogeneous and often underutilized, particularly after breast-conserving therapy or in node-negative patients, reflecting gaps between guideline recommendations and clinical practice. The development of standardized MBC-specific protocols could help ensure more consistent and evidence-based application of radiotherapy.

Adjuvant endocrine therapy remains central due to the high prevalence of ER-positive tumors. Tamoxifen significantly improves overall and recurrence-free survival, as confirmed by meta-analyses and population cohorts. However, effectiveness is limited by adherence, with 20–30% of patients discontinuing therapy within the first two years due to side effects. Aromatase inhibitors, alone or combined with GnRH analogues, appear less effective. A multidimensional approach—including close patient follow-up, targeted education, optimized side-effect management, and individualized treatment regimens—should be implemented. Digital tools, standardized follow-up consultations, and psychological support programs may further enhance adherence and clinical outcomes. Beyond adherence, men with breast cancer are also frequently diagnosed at later stages and experience delayed access to specialized care, largely due to low awareness and the rarity of the disease. Addressing these barriers will require targeted patient education, systematic follow-up consultations, digital adherence monitoring, and awareness campaigns for primary care providers to promote earlier diagnosis and timely referral. In addition, the high prevalence of AR expression strongly supports further exploration of AR-targeted therapies, potentially in combination with existing endocrine strategies. The role of systemic therapy in MBC is less well defined, as most data are extrapolated from FBC. Neoadjuvant chemotherapy may be useful for locally advanced disease, but evidence is limited to small retrospective series. Importantly, molecular information may increasingly guide treatment decisions: *BRCA2* carriers could benefit from PARP inhibitors, *PIK3CA* mutations provide a rationale for PI3K inhibition, and CDK4/6 inhibitors—already effective in ER+/HER2− FBC—are under investigation in men. Such approaches highlight the potential of integrating genomic profiling into therapeutic planning for MBC.

While current evidence highlights biological and therapeutic advances, most studies remain retrospective, underpowered, and heterogeneous, often with limited male-specific analyses. Extrapolation from FBC, though practical, risks overlooking sex-specific biology and treatment responses. Moreover, many therapeutic recommendations are based on expert consensus rather than prospective validation. A more critical perspective emphasizes that current guidelines, while useful, inadequately address the unique needs of men, and significant gaps remain in the evidence base for optimal systemic therapy, radiotherapy indications, and long-term survivorship care.

Despite these advances, several gaps persist. This review has limitations that must be acknowledged. The literature remains dominated by retrospective series, often with small sample sizes and heterogeneous reporting, precluding formal meta-analysis in most instances. While our search strategy was comprehensive, it was restricted to PubMed and PMC, which index the vast majority of relevant biomedical literature; inclusion of additional databases (e.g., Embase, Web of Science) might have retrieved further studies. To mitigate this limitation, we manually screened the reference lists of eligible articles and relevant reviews to capture additional publications. A further limitation of this review is its exclusive focus on non-metastatic disease, which, while justified by the curative potential and translational relevance of this stage, does not address the specific challenges and unmet needs of metastatic male breast cancer. Overall, the evidence base remains constrained by small sample sizes, retrospective study designs, and marked methodological heterogeneity, which limit the robustness and generalizability of current findings.

Most available cohorts remain small and often biased toward high-risk families or specific populations, limiting generalizability. Prospective male-specific studies are scarce, and included studies show considerable heterogeneity. Long-term outcome data are limited. The interactions between genetic, hormonal, and environmental factors remain poorly understood, and few studies have assessed the prognostic and predictive value of genetic and epigenetic alterations within the context of current therapies.

Our literature review provides an integrated and updated overview of the genetic, biological, and therapeutic landscape of non-metastatic male breast cancer. By synthesizing germline, somatic, and epigenetic data and contextualizing them with histopathological and clinical dimensions, we highlight MBC-specific features and the importance of considering sex differences in both research and clinical practice. The strength of this review lies in its multidisciplinary approach, linking molecular biology with therapeutic advances, and offering a coherent framework to guide future research and personalized management strategies. This integrative perspective consolidates current knowledge and paves the way for precision medicine approaches aimed at improving prognosis and quality of life for men with breast cancer.

Future research should move beyond small, retrospective series toward more collaborative and systematic approaches. A priority is the establishment of large-scale, international prospective registries that not only capture clinical data but also incorporate standardized biomaterial collection, including tumor tissue and germline DNA. Such initiatives would enable validation of prognostic biomarkers, refinement of molecular subgroups, and the design of adequately powered clinical trials tailored to MBC. These efforts are essential to bridge the gap between molecular discoveries and their translation into optimized, evidence-based management strategies.

## 5. Conclusions

Non-metastatic male breast cancer is emerging as a biologically distinct entity from female breast cancer, characterized by a genetic predisposition largely driven by *BRCA2* mutations, the involvement of moderate-penetrance genes, and a specific epigenetic architecture. This profile, combined with a predominantly luminal histological and molecular signature, highlights the need for sex-specific approaches to screening, genetic counseling, and therapeutic management.

Recent advances have demonstrated the feasibility of breast-conserving surgery followed by radiotherapy, the reliability of sentinel lymph node biopsy, and the efficacy of adjuvant endocrine therapy. However, several challenges remain, including adherence to endocrine treatment, harmonization of radiotherapy indications, and the integration of breast-conserving surgery into larger cohorts. Truly personalized care must account for male-specific anatomical features, the immunosuppressive tumor microenvironment, and individual treatment tolerance.

From a scientific perspective, future directions include the integration of multiomic data for refined risk stratification, validation of emerging biomarkers (such as FASN, ATF3, DDX3, and STC2), and the development of polygenic risk scores. These efforts should converge toward the establishment of male-specific therapeutic protocols, aimed at optimizing survival and quality of life while respecting the unique biological identity of MBC.

Despite notable progress, a major limitation persists: the historical exclusion of men from randomized clinical trials. The absence of dedicated prospective data continues to constrain clinical practice, which still relies heavily on extrapolations from FBC and expert consensus. The urgent development of clinical trials and guidelines specifically tailored to MBC is therefore imperative to align standards of care with those for women, while considering the genetic, biological, and anatomical specificities of men.

In sum, the growing understanding of the genetic and biological underpinnings of MBC, combined with therapeutic innovations, paves the way toward true precision medicine for this rare disease. This long-overlooked field now calls for collaborative and focused research, as the only path to sustainably transform both prognosis and quality of life in men with breast cancer.

## Figures and Tables

**Table 1:** Genetic predisposition to male breast cancer: penetrance, risk spectrum and associated syndrome.

					OR [IC95%]	
Gene	Chromosome	Inheritance	Pathogenic Variants	Clinical Syndrome	Pritzlaff et al. [12]	Janatova et al. [13]
High penetrance						
*BRCA2*	13q12-13	AD	LOF mutations	HBOC	13,9 (5–22)	44,0 (2–92)
*BRCA1*	17q21	AD	LOF mutations	HBOC	1,8 (0,3–6,8)	5,8 (0–14)
*TP53* *	17p13	AD	Missense/LOF mutation	Li-fraumeni	-	-
*PTEN* *	10q23	AD	LOF mutations	Cowden	-	-
*STK11* *	19	AD	LOF mutations	Peutz Jeghers	-	-
Moderate or Low penetrance						
*CHEK2*	22q11	AD	1100delC	Li-fraumeni variant	2,4 (1,3,4,9)	5,0 (1,3,8–12)
*ATM*	11q22-23	AR/AD	Truncating/missense variants	HC: breast cancer risk; BA: Ataxia–Telangiectasia	1,4 (0,1,3–5)	1,3 (0–7)
*PALB2*	16p22	AD AR	Truncating variants	HBOC Syndrome Fanconi	6,6 (1,7–21)	8,3 (2–27)

AD: Autosomal Dominant; AR: Autosomal recessive; BA: Biallelic *ATM*; HBOC: Hereditary breast and/or ovarian cancer; HC: Hetero zygous *ATM* carriers; LOF mutation: Loss of function mutation. * To date, no quantified odds ratios have been reported for *TP53, PTEN*, or *STK11* especially in male breast cancer.

**Table 2:** Histopathological and molecular characteristics of male breast cancer across selected cohorts.

Study (Reference)		Kornegoor et al. [30]	Vermeulen et al. [33]	Moelans et al. [18]	Piscuoglio et al. [20]
Histological type (%) -	Ductal Lobular Others	95 2 3	>90 <10	90 10	NR
Grade (%)	1 2 3	19 43 38	19 42 39	NR	NR
ER−/ER+	(%)	1/99	1/99	4/96	0/100
PR−/PR+	(%)	18/82	18/82	34/66	NR
HER2−/HER2+	(%)	95/5	95/5	98/2	97/3
LN−/LN+	(%)	54/46	54/46	NR	NR
Ki-67	(Low/High)	79/21	NR	NR	NR
Subtypes (%)	Luminal A like Luminal B like Basal like HER2	75 21 4 0	81 13 6 0	50 46 4 0	29 71

Abbreviations: LN = lymph nodes; NR = not reported.

**Table 3:** Survival outcomes of BCS versus mastectomy in terms of 5- and 10-year overall survival.

	First Autor (Reference)	Survival Data	
		BCS	Mastectomy
5-Year DSS	Cloyd et al. [58]	87%	88%
	Zaenger et al. [50]	100%	97.3%
5-Year OS	Den et al. [49]	84%	86%
	Zaenger et al. [50]	97%	95%
	Bateni et al. [59]	41%	40%
	Cloyd et al. [58]	66%	70%
	Fogh et al. [56]	7 of 8	34 of 34
10-Year-OS	Bateni et al. [59]	6%	5%
	Cloyd et al. [58]	47%	46%
	Fogh et al. [56]	6 of 8	23 of 34

**Table 4:** Diagnostic performance of sentinel lymph node biopsy in male breast cancer.

First Authors, Year, Source	Study Design	N (SLNB)	Identification Rate (IR)	False-Negative Rate (FNR)
Parpex, 2024 [60]	Meta-analysis, no heterogeneity	164 (IR)/50 (FNR)	99.0%	0%
Koukouras, 2012 [62]	Retrospective study	11	100%	0%
Gentilini, 2007 [63]	Retrospective study	32	100%	0%
Maràz, 2016 [64]	Retrospective study	16	100%	3/12
Lin, 2011 [48]	Meta-analysis	213	97.4%	7.4%
